# Reentry-driven model of atrial fibrillation is maintained by paired reentries and terminated by strategic pairwise virtual ablation

**DOI:** 10.3389/fphys.2025.1695431

**Published:** 2025-11-17

**Authors:** Arthur S. Bezerra, Robin Van Den Abeele, Bjorn Verstraeten, Sebastiaan Lootens, Arstanbek Okenov, Timur Nezlobinsky, Sander Hendrickx, Vincent F. M. Segers, Nele Vandersickel

**Affiliations:** 1 Department of Physics and Astronomy, Ghent University, Ghent, Belgium; 2 Department of Pharmaceutical Sciences, Antwerp University, Antwerp, Belgium

**Keywords:** atrial fibrillation, cardiac electrophysiogy, reentrant arrhythmia, topology, index theorem

## Abstract

**Introduction:**

Atrial fibrillation (AF) is a very common cardiac arrhythmia whose mechanisms are still a topic of debate. This work applied concepts of topology to gain new insights into reentry-based simulated AF, similar to our previous work in atrial tachycardia (AT). We demonstrate that the Index Theorem – which states reentries must come in pairs of opposite rotations – applies to a model of AF, even when the complex dynamics change over time. Additionally, we tested the hypothesis that connecting opposite pairs of singularities can terminate simulated AF in the same way as clinical and simulated AT.

**Methods:**

we applied a modified phase mapping capable of detecting both functional and anatomical reentry to a dataset of 600 AF simulations based on clinical data. We then compared three virtual ablation strategies: random lines, straight lines, and heuristic lines. Straight lines connected pairs of opposite singularities through the shortest path; heuristic lines connected them in such a way that prioritized blocking the conduction path; and random lines connected randomly selected pairs of points with comparable distance to the other methods.

**Results:**

We showed that our algorithm could verify the predicted paired reentries for 99% of the simulation duration on average, and 93% for the worst-performing case. The heuristic virtual ablation method terminated activity for 90% of cases, a marked improvement against the straight line method (55%) and the random method (0.5%).

**Discussion:**

This work provides mechanistic insights into AF, and points towards pitfalls of ablation strategies, both of which have the potential to improve our understanding and ability to treat this condition.

## 1 Introduction

The mechanisms behind atrial fibrillation (AF), the most common cardiac arrhythmia, are still not fully understood. Here, we apply concepts from topology to reentry-based simulated AF, expanding our recent works in which a similar methodology brought new mechanistic insights into atrial tachycardia (AT) (Duytschaever et al., 2024; Abeele et al., 2025).

In the context of cardiac electrophysiology, phase mapping associates the different states a cardiomyocite or patch of cardiac tissue may assume to different values in a limited range. Typically that range is [−π,π] , with the extremes being associated to resting and excited tissue. There are multiple methods to obtain such a mapping, but they have general properties that are independent of the particular choice (Arno, 2021; Lootens et al., 2024).

For a given phase field ϕ , the index, also known as the topological charge (TC) , associated with a curve C in space is defined in Equation 1. Being a closed integral over a gradient field, the index will typically be 0, but when a discontinuity in the field is present, it may assume non-zero values. Since mapping is typically chosen so that phase discontinuities correspond to wavefronts, an alternative way to calculate the phase index is to simply count the number of times a wavefront crosses the curve C , adding −1 for clockwise crossings and +1 otherwise (Arno, 2021; Lootens et al., 2024).

$$TC\left(C\right)=\frac{1}{2\pi }\oint_{C}\vec{\nabla }\phi \cdot \vec{d\ell }$$ (1)

A phase singularity, or singular point, is defined as a point around which any sufficiently small curve C will yield a non-zero value for TC . Phase singularities are commonly associated with rotor tips, but more generally are associated with the endpoints of wavefronts, as shown by Marcotte et al. (Marcotte and Grigoriev, 2017). Finally, the Index Theorem states that the total index associated with a closed surface must always be zero, meaning that the total number of positive and negative singularities must be equal.

In our previous works, we extended the concept of phase singularities, showing that the Index Theorem holds not only for singular points, but also when integrating around anatomical boundaries. As a consequence, anatomical reentries bounded to a two-dimensional surface must always come in pairs of opposite chirality, represented by an index of +1 or −1 (Duytschaever et al., 2024; Abeele et al., 2025; Vandersickel et al., 2023; Vandersickel, 2024; Duytschaever et al., 2025).

This fundamental property had been previously overlooked, largely due to the presence of incomplete reentries–circuits that fail to complete a full rotation before colliding with another wave. Historically, these passive reentries were considered unimportant for the maintenance of AT.

However, we demonstrated that an incomplete reentry–sometimes called a passive, bystander, or wannabe reentry (Lee et al., 2015; Maury et al., 2019) – is just as critical for sustaining AT as a complete (or full, driving, active) reentry. Although it does not complete a full rotation in physical space, the line integral around it still results in a non-zero value, describing a full rotation through phase space. Consequently, if ablation only targets the more easily identifiable full circuits while neglecting incomplete ones, arrhythmia will not terminate, and the incomplete circuit may even evolve into a complete one. Therefore, both complete and incomplete reentries must be ablated to achieve termination. Due to the Index Theorem, each clockwise rotation is matched by a counterclockwise counterpart, which may be of either type.

**FIGURE 1:**
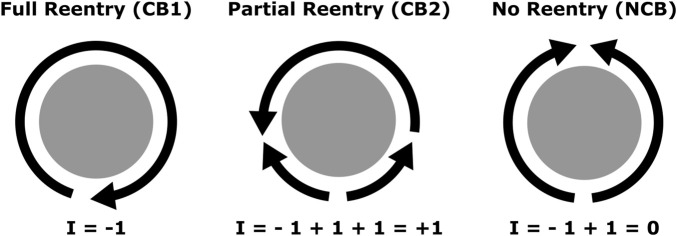
Diagram representing the previously described interactions between propagation waves (arrows) and a boundary (in gray). Each arrow’s head marks a wavefront, and its trailing line marks a waveback. From left to right, CB1 (complete reentry), CB2 (incomplete reentry), and NCB (no reentry). Each wavefront contributes to the index by a factor of +1 or −1 depending on direction.

Based on this understanding, we classified anatomical boundaries between 3 types: critical boundary type 1 (CB1), which hosts a complete reentry; critical boundary type 2 (CB2), which hosts an incomplete reentry; and non-critical boundary (NCB), which does not host a reentry. Despite clinical terminology often referring to complete reentries as “active” or “driving” reentries (Santucci et al., 2024), any combination of CB1 and CB2 pairs may be present in a given case, depending on geometry and distribution of conduction velocity values (e.g., CB1-CB1, CB1-CB2, CB2-CB2). Regardless, all CBs must be connected by an ablation line to a CB of opposite index in order to terminate the arrhythmia. All three categories are illustrated in Figure 1. Note that multiple waves may interact with the same boundary, and the net total in each direction will determine the corresponding index, as described previously.

Now, we seek to verify the Index Theorem for simulated AF, and explore its mechanistic implications. A commonly proposed AF mechanism is the presence of not only anatomical reentry but also functional reentry (rotors). According to the Index Theorem, singularities must come in pairs, including both functional and anatomical reentries. Paired rotors of opposite chirality have been described in multiple studies (Xu et al., 2023; Atienza et al., 2015; Gurevich and Roman, 2019). However, paired rotors are often described as a mere alternative to single rotors, which are considered an equally valid configuration (Lin et al., 2013; Narayan et al., 2014; Nattel et al., 2017; Rappel et al., 2024). That is likely because those works did not consider other types of singularity these rotors may be paired with.

In theoretical works focused specifically on singularity topology, it has been demonstrated that the topology of electrical propagation requires that phase singularities are only created or destroyed in counter-rotating pairs (Marcotte and Grigoriev, 2017; DeTal et al., 2022; Arno et al., 2024). They show that the total sum of the index, or topological charge, is a conserved quantity, analogous to the Index Theorem. However, boundary interactions were either not analyzed, or treated as a problem that can break this conservation. This limitation weakens the value of this topological approach, as wave collisions with boundaries are common.

Here, we reconcile our previous work, where rotation around boundaries is shown to obey the Index Theorem, with the above studies, which overlooked the contribution of boundaries when calculating the total topological charge. Topologically, an anatomical reentry is equivalent to a non-meandering rotor, as the boundary can be continuously reduced to an arbitrarily small core, like that of a rotor. Alternatively, the refractory core around which a rotor is anchored, or around which it meanders, can be treated identically to an anatomical reentry (Arno et al., 2024; Tomii et al., 2021). Moreover, rotor tips are topologically equivalent to other phase singularities with no special name, associated to the endpoints of wavefronts (Marcotte and Grigoriev, 2017) and non-excitable cores (Arno et al., 2024; Tomii et al., 2021). Any singularity has the potential to become a rotor tip, depending mainly on its interactions with other waves and refractory tissue, and whether those interactions result in sustained reentry or other behavior, such as meandering.

Therefore, as we have already showed in previous works that the Index Theorem holds for regular, anatomical reentries, and there is a topological equivalence between anatomical and functional reentries, it is to be expected that it also will hold for irregular, functional reentries–which we investigate in this work.

This work aims to show that the Index Theorem holds for simulated AF data that includes a mix of functional and anatomical reentries, often with unstable activity, waves breaking and merging, and boundary interactions. Furthermore, we show that the identification of singularities is fundamental for AF termination through virtual ablation. As the previous related works (Marcotte and Grigoriev, 2017; DeTal et al., 2022; Arno et al., 2024) were more concerned with the theoretical aspects, they only show a small number of examples, which were not anatomically realistic and did not consider boundary effects. To demonstrate it at a larger scale, we performed 600 simulations based on a previously described set of 100 anatomically modeled 3D meshes, which include realistic fiber direction, MRI-based fibrosis levels, and remodeling. Moreover, expanding on our previous work, we compare different ablation methods, obtaining unique mechanistic insights into the dynamics of irregular activity.

## 2 Materials and methods

To demonstrate our concepts, we generated a set of 600 AF simulations, from which we extracted the phase from the action potential signals. For each time-step, we identified and clustered phase singularities. Additionally, for select points in time, we used a modified distance based on the phase value to consider possible ablation lines between opposite-chirality cluster pairs; and finally, applied the combination of ablation lines that minimized the total ablation length. As a baseline for comparing outcomes, for each simulation we also generated a set of geodesic lines connecting opposite-chirality pairs, and a set of random ablation lines with approximately the same length.

### 2.1 Simulated data

Single Cell Model: All simulations were executed using the OpenCARP simulation software (Plank et al., 2021). We used the Courtemanche ionic model with AF remodeling as the electrophysiological model of our simulations (Courtemanche et al., 1998). This remodeling encompasses a reduction of the ultra-rapid delayed rectifier conductivity $\left(G_{\mathrm{Kur}}\right)$ by 50%, a reduction of transient outward potassium $\left(G_{to}\right)$ by 50% and a reduction of 70% to the L-type calcium conductivity $\left(G_{\mathrm{CaL}}\right)$ .

Rather than modeling fibrotic regions as non-conductive tissue interspersed with conductive tissue, they were treated as conductive tissues with a further adapted ionic model. $G_{\mathrm{Kur}}$ was reduced by 50%, $G_{to}$ by 50%, $G_{\mathrm{CaL}}$ by 50% and $G_{Na}$ was reduced by 30%. These values were based on the ones used by Roney et al. (2022), with the exception of the $G_{Na}$ value, which was changed from 60% reduction to 30% reduction. The original value led to unstable simulations that rapidly terminated, whereas the adjusted value resulted in more stable, longer-lasting simulations. Although less often, spontaneous termination still occurred, which we will address later.

These parameter changes resulted in a 14% reduction of CV relative to non-fibrotic regions. $APD_{90}$ values were measured with a pacing frequency of 2 Hz, resulting in 194 ms for general remodeled tissue and 233 ms for fibrotic tissue, close to the values reported by Courtemanche et al. for the AF-remodeled tissue (Courtemanche et al., 1998).

Transmembrane voltages were recorded in 5 ms time-steps (200 Hz sampling frequency).

Mesh parameters: For our simulations, we used a publicly available dataset by Roney et al. (2022) consisting of 100 3D meshes of left atria based on clinical AF data, including MRI intensity and fiber orientations. It included 43 paroxysmal, 41 persistent, and 16 long-standing persistent AF patients, none of which had previously gone through ablation treatment. The meshes were refined to achieve an average edge length of 160–170 μm for better simulation precision. To mimic fibrosis, different conduction velocity values were assigned based on MRI intensity, as described in Table 1. Conduction velocity was then further reduced by 50% in the directions orthogonal to the fiber orientation.

**TABLE 1:** Conduction velocity and model version by MRI intensity (IIR).

MRI	Conduction	Model
Intensity	Velocity (m/s)	Version
IIR<0.9	0.81	Global model
0.9<IIR<1.1	0.76	Global model
1.1<IIR<1.22	0.72	Fibrotic model
1.22<IIR<1.4	0.68	Fibrotic model
1.4<IIR<1.6	0.63	Fibrotic model
1.6<IIR	0.58	Fibrotic model

**FIGURE 2:**
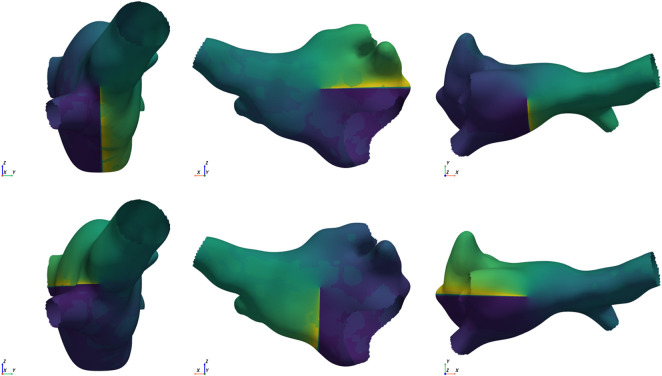
Example of the 6 starting conditions for the same mesh. Singularities in opposite directions are placed along each of the Cartesian axes (from left to right: x, y, and z axis). The top row shows the clockwise placement, and the bottom row shows the counter-clockwise placement.

Reentry induction: On each mesh, we induced reentry by means of phase singularity distribution. We distributed phases from - π to π around each Cartesian axis, once in clockwise and once in counter-clockwise direction, resulting in a total of 6 different starting conditions for each mesh, illustrated in Figure 2. Then 5 pre-pacing beats were applied before starting the simulation up to 3,400 ms.

All simulations were analyzed starting from time = 50 ms until the final time of 3,400 ms, or until early termination, whichever happened first. A simulation was considered to terminate early, i.e., to spontaneously terminate, if all activity ceased without the need of any ablation. The termination time was defined as the moment after which all transmembrane voltages remain below −40 mV.

### 2.2 Phase singularity detection

While a fine-resolution 3D mesh was necessary to obtain accurate dynamic behavior for simulations, our subsequent analysis could be performed with coarser resolution, reducing computation times significantly. Therefore, apart from simulations, all analysis was performed on a subsampled version of the mesh in order to obtain approximately even-spaced points at a distance of 2 mm from each other.

**FIGURE 3:**
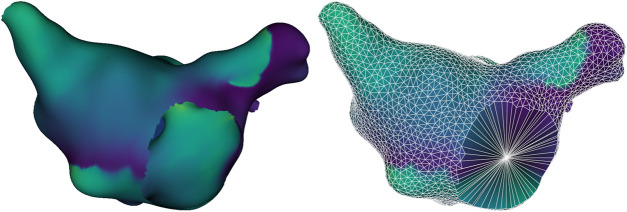
Example of an original simulation mesh (left) and reduced mesh (right). The reduced mesh displays the geodesic Voronoi diagram connecting neighboring sampled points with white edges, and includes an additional point at the center of each anatomical hole, allowing the identification of anatomical reentries around it.

To identify phase singularities, we applied a modified phase mapping method. We obtained local activation times (LATs) from the transmembrane voltages of the sampled points, and calculated their phase ϕ using the sawtooth technique, in the range of [−π,π] (Lootens et al., 2024). In order to detect anatomical reentries, an additional point was added at the center of each anatomical cavity (mitral valve and the four pulmonary veins). A Voronoi diagram based on geodesic distances was used to determine which sampled points were geometrically neighbors, including the additional points from the anatomical cavities, as illustrated in Figure 3. Formally, anatomical reentries are not singularity points, but act as a distributed phase discontinuity of finite size, which can be continuously deformed into a single point as previously described. Therefore, they were treated in the same way for the purposes of our calculations, unifying our previous AT work and singularity-based phase mapping.

As explained in the Introduction, counting the number of discontinuities caused by the presence of wavefronts is a simple way to obtain the phase index. To do so, each point’s neighbors were first sorted in counterclockwise order, and phase differences between consecutive neighbors were computed. A phase difference was classified as a *phase jump* if |Δϕ|>π . For each time step, phase jumps in the neighborhood of a point were counted, adding +1 to that point’s index for counterclockwise jumps and −1 for clockwise jumps. This procedure is formalized in Equations 2, 3, where the topological charge TC(p) of point p is derived from the phase differences between consecutive points i and j in its sorted neighborhood $N_{p}$ .

$$TC\left(p\right)=\underset{i,j\in N_{p}}{\sum }J\left(\Delta \phi_{ij}\right)$$ (2)

$$J\left(x\right)=\left\{\begin{array}{cc} -1\; & \text{if }x<-\pi \\ 1\; & \text{if }x>\pi \\ 0\; & \text{otherwise} \end{array}\right.$$ (3)

Points with an unequal number of phase jumps in each direction had a nonzero index and were tagged as singularities at that time step. This definition accommodates multiple jumps in each direction. In the terms of our previous AT works (Duytschaever et al., 2024; Abeele et al., 2025), a complete circuit (CB1) will have just one phase jump for its entire duration, while an incomplete circuit (CB2) will have two jumps occurring in one direction and one jump in the other for some of its duration. It is also possible for a boundary to have index values >1 , indicative of multiple jumps in the same direction. Due to the inclusion of additional points within cavities, both functional and anatomical reentries were detected simultaneously and treated identically.

After being identified, singularities were clustered, first in space and then in time. At each timestep, given the mean distance between neighbors in the subsampled mesh $\left(\mu_{d}\right)$ , DBSCAN clustering with $\epsilon =2\mu_{d}$ was applied separately to the positive and negative singularities. Then, between each time-step and the next, clusters of same chirality were merged if they were within the same distance threshold ϵ .

This approach allowed us to identify which detections belonged to the same singularity and to measure each cluster’s lifespan, accounting for meandering. In all metrics described below, each cluster was treated as a single singularity rather than multiple detections. For example, a cluster of three points with index of −1 each, only contributed −1 to the total index, not −3.

#### 2.2.1 Adherence to the Index Theorem

We defined two performance metrics based on our algorithm’s ability to detect singularities and track them over time–and therefore they are measures of the algorithm’s adherence to the expected results for the Index Theorem.

They are two relative measures: $f_{0}$ , the fraction of the simulation time in which the index sum was 0; and $m_{\mathrm{ind}}$ , the mean index sum over the simulation time. Being relative measures, they allow a consistent way to directly compare different cases, since early termination leads to some simulations lasting less than others.

#### 2.2.2 Arrhythmia complexity

To verify that our simulations were suitably complex for AF, we estimated how long singularities lasted, the total number of singularities over time, and the highest number of simultaneous singularities. Unlike the Index Theorem consistency measures, we did not make a distinction between positive and negative singularities here. Between these three measures, we can observe for each simulation the stability of its singularities, how often they tended to be created and destroyed, and the largest number of simultaneously hosted singularities.

For a more clinically grounded perspective on the data, the cycle length (CL) median and interquartile range were calculated for all simulations and compared to typical clinical values. The activation times of each vertex were calculated from the transmembrane voltages, and the differences between consecutive activations obtained. Then, using the distribution of activation time differences for a simulation, the median and interquartile range–the difference between the 75th and 25th percentiles–were calculated.

#### 2.2.3 Comparison of starting conditions

Since each of the 100 meshes were simulated under 6 different starting conditions, it is relevant whether there were relationships between simulations originating from the same mesh. Therefore, we recorded how often the simulations from each mesh terminated early and whether singularity hot spots were spatially correlated between different starting conditions for the same mesh.

To check for singularity hot spots for a given simulation, we calculated the singularity count $S_{c}\left(p\right)$ of each point over time, as per Equation 4, where TC(p,t) is the topological charge of point p at time t , and $t_{f}$ is the final simulation time. Note the metric does not take rotation direction into account, only the presence of a singularity.

$$S_{c}\left(p\right)=\overset{t_{f}}{\underset{t=0}{\sum }}|TC\left(p,t\right)|$$ (4)

To compare the distribution of $S_{c}\left(p\right)$ values between different simulations originating from the same mesh, we used the global bivariate Moran’s I , a modified correlation that takes spatial information into account (Wartenberg, 1985). It is defined in Equation 5 where x and y are two scalar fields over a discrete set of points; $\bar{x}$ , $\bar{y}$ , $\sigma_{x}$ and $\sigma_{y}$ are their means and standard deviations respectively; and $w_{ij}$ is a kernel that provides spatial information between points. The kernel chosen in this work is described by Equation 6, where $d_{ij}$ is the geodesic distance between points i and j , and R=2mm is the sampling radius. It was chosen to measure distribution similarities between neighboring points, rather than strict point-to-point correspondences.

$$I_{M}\left(w,x,y\right)=\frac{\sum_{i}\sum_{j}w_{ij}\left(x_{i}-\bar{x}\right)\left(y_{j}-\bar{y}\right)}{\sigma_{x}\sigma_{y}\sum_{i}\sum_{j}w_{ij}}$$ (5)

$$w_{ij}=\left\{\begin{array}{cc} 0\; & \text{if }i=j\\ e^{-{\left(\frac{d_{ij}}{2R}\right)}^{2}}\; & \text{otherwise } \end{array}\right.$$ (6)

$I_{M}$ is not necessarily bounded within the [−1,1] interval like a common Pearson correlation, and the spatial self-similarity measured by $I_{M}\left(x,x\right)$ is not necessarily equal to 1, as the scalar field x may have significant variation between nearby points. To provide a meaningful reference, we calculated both the values of Moran’s I within the same simulation (self-similarity) and between different simulations created from the same mesh (cross-similarity). If cross-similarity values are comparable to self-similarity values, a stronger role of the substrate and geometry can be inferred; otherwise, a stronger role of initial conditions can be assumed.

### 2.3 Ablation strategy

To obtain mechanistic insights on the effects of ablation in our simulations, we generated virtual ablations. These are simulated lines of conduction block created instantaneously, idealized approximations of a real ablation. Different strategies were used to determine the size, shape, and position of each virtual ablation.

Heuristic lines: Virtual ablation lines were created for each simulation at the points in time of 1,000 ms, 2000 ms, and 3,000 ms. At each time-step, points within each cluster were treated as one singularity, and if a cluster contained multiple points, a conduction block loop was created passing through all of them. Virtual ablation lines were then created to connect pairs of opposite singularities while blocking the wave path and minimizing virtual ablation length. If there was a detection issue that caused an unequal number of opposite singularities, we iteratively increased the detection time-step by 5 ms until the index sum was 0.

To create a virtual ablation line between a pair of opposite-chirality singularities, the Dijkstra traversal algorithm was used to find the lowest-cost path between the two points. In order to obtain the desired effect of blocking wave paths while minimizing distance, a heuristic distance $d_{\mathrm{heur}}$ was used as the edge weight for the Dijkstra algorithm. The heuristic combines the Euclidean distance $d_{\mathrm{euc}}$ between neighboring points and their current phase values ϕ , as per Equation 7.

$$d_{\mathrm{heur}}\left(x,y\right)=\frac{d_{\mathrm{euc}}\left(x,y\right)}{\left(\phi \left(x\right)+1.1\pi \right)\left(\phi \left(y\right)+1.1\pi \right)}$$ (7)

As ϕ∈[−π,π] , the term of 1.1π ensures all phases are strictly positive, reducing the heuristic distance for resting tissue about to be activated by the wavefront–prioritizing paths in front of the wavefront–and increasing it for tissue that was just activated–penalizing paths through those areas. Because phases change over time, the heuristic distance between the same two points also evolves dynamically. Anatomical boundary centers are an exception: as they do not have defined phases, they were set to $d_{\mathrm{heur}}=0$ for all their neighbors, additionally prioritizing paths that pass through them.

The path calculation was repeated for each possible combination of opposite-chirality singularities, and the set of combinations that resulted in the smallest total virtual ablation (in heuristic distance) was chosen and performed. Because of the heuristic, these virtual ablations tend to be between singularities that belong to the same wavefront, but on occasion may connect points from different waves–for instance, if the distance between them is small enough to overcome the penalty to not ablating at the wavefront. Therefore, the method prioritizes blocking waves directly, but allows forcing collisions between different waves when singularities are sufficiently near each other.

**FIGURE 4:**
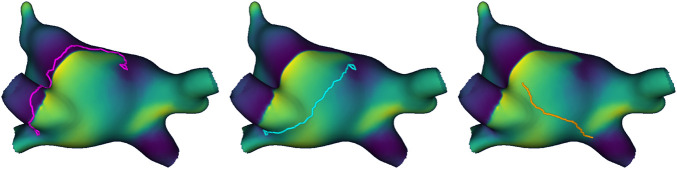
Example of the three types of virtual ablation line, from left to right: heuristic line (pink), straight line (blue), and random line (orange). Both the heuristic line and straight line form a loop around the same singularity detections and then connect them, but the former blocks the path of the wave, while the latter takes the shortest path. The random line connects the shortest path between two random points at a comparable distance to the other virtual ablation lines.

Straight lines and random lines: As baselines to compare the heuristic virtual ablation lines, we generated two other types of virtual ablation lines for each simulation at the designated virtual ablation times: straight lines and random lines. In the straight line method, we connect singularities using the same logic described above, but using the geodesic distance over the surface rather than the heuristic distance–the shortest geometric path in the manifold of the reduced mesh. In the random method, we generated straight lines of comparable size to our heuristic virtual ablations, but connecting random pairs of points rather than singularities. To do so, we calculated the geodesic distance between all pairs of points in the subsampled mesh, and divided the pairs into distance deciles (10-percentile bins). For each singularity-based virtual ablation line, a new pair was randomly selected from the same distance decile as the corresponding original pair, assuring the random lines have comparable size. Figure 4 illustrates the three types of virtual ablation lines.

Once a virtual ablation line was created using the subsampled mesh, it was translated back into the full mesh by finding geodesic paths between consecutive sampled points. This way, the state of the simulation at the moment of virtual ablation could be re-created, and block lines placed instantly at the corresponding point in time. A virtual ablation was deemed successful if all activity ceased 400 ms after creation of the block line. For the virtual ablation simulations, transmembrane voltages were recorded in 10 ms time-steps (100 Hz sampling frequency).

#### 2.3.1 Spontaneous termination

Virtual ablations were still performed on simulations that terminated spontaneously, but no virtul ablation was performed if one of the virtual ablation times (1,000 ms, 2000 ms, 3,000 ms) was after the termination time, as there would be no effect. However, virtual ablation was still performed for times before the termination time–e.g., if the early termination time was 2,500 ms, the virtual ablations at 1,000 ms and 2000 ms would be performed, but not the one at 3,000 ms. We recorded which simulations terminated early, what their termination times were, and how virtual ablation affected termination time.

Additional criteria were required to determine virtual ablation success for spontaneous termination cases. Each outcome was simulated from the moment of virtual ablation $t_{\mathrm{abl}}$ to an endpoint 400 ms later, $t_{\mathrm{end}}=t_{\mathrm{abl}}+400$ . Calling the spontaneous termination time $t_{sp}$ , and the ablation-induced termination time $t_{ai}$ we have the following criteria:

- If $t_{sp}<t_{\mathrm{abl}}$ : No simulation was performed, as there would be no activity to terminate. There is no $t_{ai}$ , as no virtual ablation is performed.
- If $t_{\mathrm{end}}<t_{sp}$ : The virtual ablation was a success if it caused termination within the window, $t_{\mathrm{abl}}<t_{ai}<t_{\mathrm{end}}$ .
- If $t_{\mathrm{abl}}\leq t_{sp}\leq t_{\mathrm{end}}$ : The virtual ablation was a success if it caused termination at least one time-step (10 ms) before the spontaneous termination, $t_{\mathrm{abl}}<t_{ai}\leq t_{sp}-10$ .

## 3 Results

### 3.1 Representative examples

**FIGURE 5:**
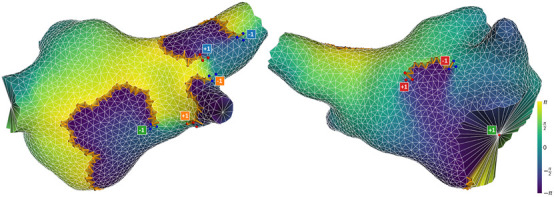
Snapshot of a successful detection, viewed from different angles. The mesh is colored according to phase values. Phase jump detections are marked with orange arrows in the direction of propagation. Detections of positive and negative singularities are marked by red and blue dots respectively. A label is placed next to each cluster of points, indicating that cluster’s contribution to the total index, and matching the color of the opposite-direction singularity associated with the same wavefront. Note that the pair marked in green is an anatomical reentry (mitral valve) paired with a functional reentry.

Before showing our general results for the dataset, we illustrate the TC detections and clustering with some representative examples. Figure 5 shows a snapshot of a case in our dataset where the index sum was 0 for its entirety, at a time when there were 4 positive and 4 negative singularities. The color-coding highlights how each wavefront is associated with a pair of opposite singularities, and that these pairs may be either functional-functional or functional-anatomical.

**FIGURE 6:**
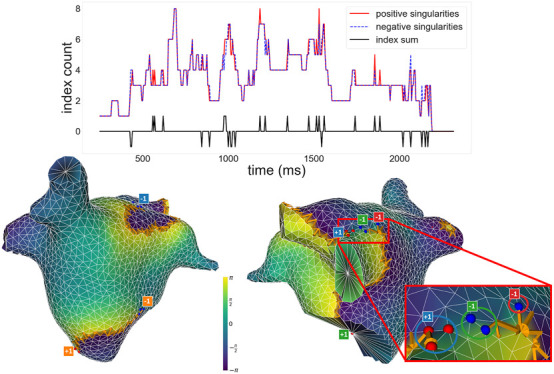
Top: Plot of the number of positive and negative singularities (solid red line and dashed blue line, respectively), and the index sum (solid black line), over time for a given simulation. Positive and negative singularities vary over time, but the index sum remains 0 for the majority of the time, with small fluctuations. Bottom: Snapshot of a moment when the index sum did not equal 0, viewed from different angles and represented with the same conventions as Figure 5. To the right, the image zooms into a section of the simulation where an incorrect detection occurred. Circles highlight which singularity detections were assigned to which clusters in the zoomed section. An unpaired −1, marked in red, results in a non-zero total index.

Figure 6 (top panel) illustrates the time evolution of the number of singularities and index sum for our worst-performing simulation in terms of the measure $f_{0}$ , while the bottom panel shows a snapshot illustrating how the failure occurred. As expected, the number of positive and negative detections varies over time, but remain equal to each other for most of the simulation. Therefore, the total index sum largely remains =0 . The detection can make mistakes, but they last only a few time-steps, and for the majority of the time evolution, a net index of zero is observed–with as few as 1 singularity of each type, and as many as 8 of each type, the red line and the blue line largely overlap, following each other closely.

To demonstrate the adherence to the Index Theorem, we used $f_{0}$ , the fraction of the total time where the index sum equals 0; and $m_{\mathrm{ind}}$ , the mean index sum over time. In this example, it is clear that the index sum is 0 for the majority of the time, and the detection errors slightly skew towards positive index sums. This is reflected on the values of our measures for this case, $f_{0}=0.9275$ and $m_{\mathrm{ind}}=0.005$ , meaning the index sum was not 0 for 7.25% of its duration. Notably, this is an early termination case, and therefore was only analyzed up to its estimated termination time of 2,320 ms, meaning the 7.25% translates to approximately 168 ms. However, the exact termination time may vary depending on our choice of transmembrane voltage threshold, hence why the counts of positive and negative singularities go to 0 a few time-steps before the end of the analysis window.

The bottom panels of Figure 6 show a snapshot where the index sum did not equal 0 for that simulation. Note how most singularities are paired off as expected, with a wavefront associated to each pair, but an extra −1 (red) remains unpaired–associated with a wavefront that already contains a pair (green). The area near a wavefront has typically high phase differences well above π , but differences are sometimes lower, and the wavefront can be disjointed in parts: here, a single missing phase jump near the wavefront causes the detection of the extra −1 . A few adjustments could avoid the issue: a lower phase jump threshold would have detected a missing part of the wavefront and not detected the red −1 ; and a higher threshold would have truncated the wavefront, not detecting the green −1 . Alternatively, maintaining the phase jump threshold, the red −1 and green −1 could have been joined into one cluster if the clustering distance parameter was adjusted. Notably, these errors, as well as all snapshots not shown here, were simple algorithmic issues, instead of failures of the Index Theorem. Small parameter adjustments could solve these problems, such as lowering the phase jump threshold, or the clustering distance, but that would cause similar problems in other simulations with different geometries or phase distributions. Rather than overfit the parameters, we acknowledge these issues here and present the results as such.

### 3.2 Phase singularity detection

#### 3.2.1 Adherence of the Index Theorem

**FIGURE 7:**
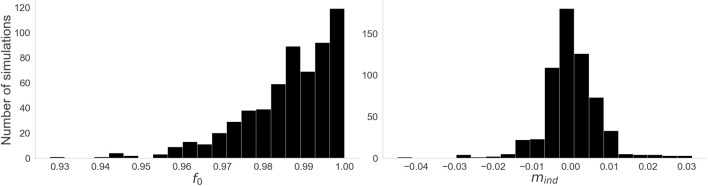
Histograms for the two performance measures. Left: $f_{0}$ , the fraction of the total time where the index sum adds up to 0. Right: $m_{\mathrm{ind}}$ , the mean index sum over time. Both measures were consistent with the Index Theorem, $f_{0}$ heavily skewing towards 1, and $m_{\mathrm{ind}}$ narrowly centered around 0.

Figure 7 shows histograms of our two performance measures across all simulations of the dataset. All simulations had a very high adherence to the expected Index Theorem result, with the lowest value of $f_{0}=0.9275$ , and a median of $f_{0}=0.9889$ . Additionally, all simulations were in the range of $-0.045\leq m_{\mathrm{ind}}\leq 0.032$ , and 89% were in the range $-0.01\leq m_{\mathrm{ind}}\leq 0.01$ .

#### 3.2.2 Arrhythmia complexity

**FIGURE 8:**
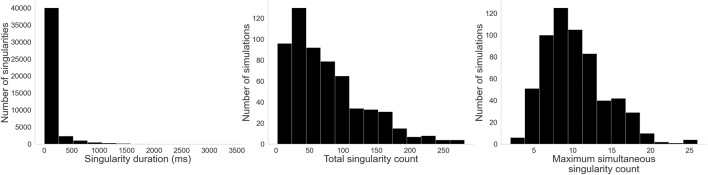
Histograms for the three complexity measures: Left: Singularity duration. While a few singularities last for most of the simulation, by far most of them are rather short-lived. Center: Total singularity count over time. As singularities are created and destroyed through the evolution of each simulation, many singularities can be detected. Right: Maximum simultaneous singularity count. Unlike AT, where only two counter rotating reentries are typically observed, here we see up to 26 singularities co-existing in some cases.

Figure 8 shows the distribution of values for our three complexity measures. On the left, we see that the majority of singularities are very short-lived, with a median value of 30 ms. However, some outliers are significantly more stable, persisting for most of the simulation–in one instance, a singularity lasted for all 3,400 ms of the simulation. On the center, we have the distribution of total singularity counts for the entire duration of each simulation. While the most stable cases had only 2 singularities for their entire duration, in the majority of cases many singularities are created and destroyed over time: a median count of 62 singularities over the simulation, and some cases reaching upwards of 250. On the right, the distribution of maximum simultaneous singularities shows again that the simplest cases had only 2 simultaneous singularities, but the median case had 10 and some had up to 26.

**FIGURE 9:**
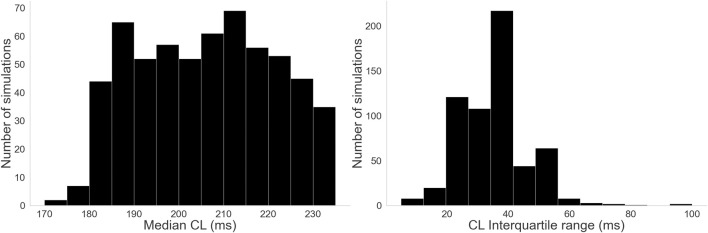
Histograms for the three complexity measures: Left: Distribution of median CL values. Right: Distribution of CL interquartile range values.

Figure 9 shows the distribution of values for our CL measures. Clinical AF CL values range from around 90 ms–250 ms and are typically centered around 180 ms (Haïssaguerre et al., 2004; Nagy et al., 2021). Our distribution of median CL, while within that margin, leans towards slower values. However, the spread associated with the interquartile range suggests some cases had significantly faster regions or moments of activity, consistent with the irregularity of AF.

#### 3.2.3 Comparison of starting conditions

**FIGURE 10:**
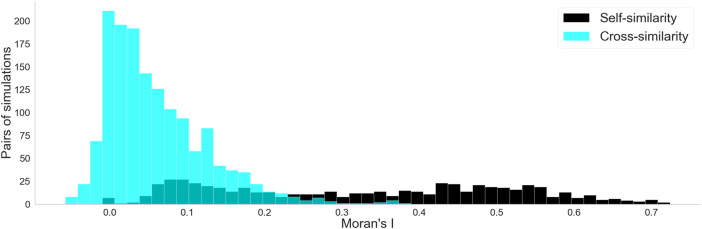
Histograms for the two Moran’s I distributions: the self-similarity (black) and the cross-similarity (blue). The self-similarity has a higher median and higher variance.

For each mesh, we calculated the Moran’s I between the singularity counts of the different starting conditions (cross-similarity), and of each given starting condition with itself (self-similarity). Figure 10 shows the distribution of the values for each. The self-similarity values were spread over a range of values from 0 to 0.72, with a median of 0.33. The cross-similarity values ranged from −0.05 to 0.38, with a median of 0.04. As the exact range of Moran’s I depends on the distributions and chosen kernel, the main relevance in these values is in their comparison: while there was some overlap between the distributions, the cross-similarity is much less spread out and has a much lower median compared to the cross-similarity. This indicates an overall low similarity between different starting conditions for the same mesh.

#### 3.2.4 Spontaneous termination

**FIGURE 11:**
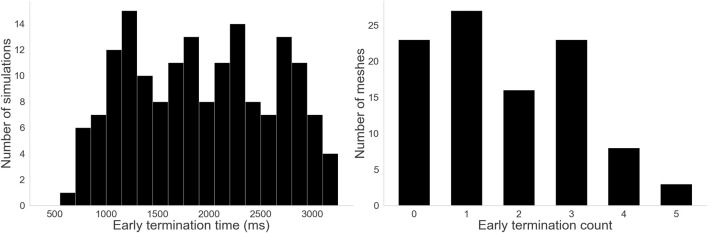
Histograms for the two termination measures. Left: Distribution of early termination times. A wide variety of termination times were observed, with some lasting almost the full duration of the simulation and some terminating before even the first virtual ablation time. Right: Distribution of early termination counts–how many starting conditions terminated early per mesh. 23 meshes never terminated early, and only 3 terminated early for 5 out of 6 starting conditions. Other meshes fell somewhere between, but notably no mesh terminated early for all 6 starting conditions.

29% of the dataset spontaneously terminated. Figure 11 shows on the left the distribution of early termination times across those cases, with a median of 1952 ms. Figure 11 shows on the right that depending on the mesh, anywhere between 0 and 5 out of 6 starting conditions resulted in early termination, though most meshes leaned towards not terminating early. Notably, all meshes had at least one starting condition that could sustain activity for the full duration rather than spontaneously terminating.

### 3.3 Virtual ablation outcomes

As previously described, virtual ablations were not performed when a case spontaneously terminated before the chosen virtual ablation time, resulting in a total of 1,617 simulations per virtual ablation strategy (out of a possible 1800, had no early terminations occurred). Following the success metrics for each of the virtual ablation strategies, we separately calculated the performance for the spontaneous termination cases and for the non-spontaneous termination cases, as well as the overall performance, as seen in Table 2. The results are consistent between the two groups, except for a minor increase in the very low performance of random lines. The straight lines showed a marked improvement compared to the baseline of the random strategy, but still failed roughly half the time. The heuristic lines on the other hand, almost always succeeded, though they performed better for the non-spontaneous cases.

**TABLE 2:** Success rates across virtual ablation strategies, grouped by termination type.

Virtual ablation	Non-spontaneous	Spontaneous	Full
Strategy	Termination	Termination	Dataset
Random lines	0.004	0.009	0.005
Straight lines	0.573	0.421	0.549
Heuristic lines	0.920	0.778	0.899

## 4 Discussion

### 4.1 Paired rotations and ablation in AF

In this work, we extended our previous results from AT to simulated AF, demonstrating how the Index Theorem holds for 600 patient-based AF simulations. Note that algorithmic failures to detect an index sum of 0 do not suggest that the Index Theorem is not true: they show that even with an imperfect algorithm, it is possible to observe its adherence for the majority of the time, reinforcing its relevance for AF dynamics. We therefore generalize our previous results for both functional and anatomical reentries, as well as irregular activity.

We also showed that opposite-chirality singularities must be connected to terminate the arrhythmia. However, we observed that only connecting the singularities with a block line is often insufficient, and that the particular way that these connections are made plays a major role. The necessity of connecting all singularities suggests that arrhythmic activity is sustained not only by long-lived anatomical reentries and rotors, but also by shorter-lived singularities, as ignoring them during virtual ablation may lead to continued activity.

As the heuristic method was designed to block the wave paths, its high performance is expected, and its significantly higher value compared to the straight line method highlights the significance of the particular way singularities are connected by ablation lines–in contrast to our previous AT work, where simply connecting pairs of CBs was sufficient. The heuristic method was a “hard limit”, in which the path was completely blocked; but less aggressive strategies that still block conduction paths and do not allow reoccurrence may be possible.

Observing the 8% of failed heuristic virtual ablations in cases that did not terminate early, we noted that all of these cases had something in common: despite being designed to do so, the conduction block lines did not fully block the waves at the moment of virtual ablation. This arises from the specific design of our heuristic, which weighs paths between points using a combination of their geometric distance and phase values at that point in time. Depending on the relative sizes and positions of different waves, these two weight components may act in opposition: large geometric distances increase the weight, whereas close phase alignment with a wave decreases it. In such cases, the minimum-weight path may connect singularities from different waves, thereby failing to block the waves as intended. The fact that this happened in every case where the heuristic lines failed strongly points towards the idea that those failures were caused by improper connection of singularities, again suggesting that very particular forms of ablation are required to truly terminate the arrhythmic activity.

As supporting results, we also analyzed the simulation dynamics, showing that the simulations were fittingly complex for AF, outside of a small number of outliers. This was a particularly relevant concern due to the relatively artificial starting conditions, based on phase singularity placement. However, as we observed, tens or even hundreds of singularities were created or destroyed per simulation. Most of these singularities were rather short lived, and most cases hosted multiple of them simultaneously, demonstrating the evolution away from the simplified initial setup. Almost 30% of simulations terminated spontaneously, but all meshes had at least one starting condition that did not. Notably, not only was inducibility greatly affected by the starting conditions, but also very little spatial correlation was found between singularity hot spots from different simulations on the same mesh–possibly suggesting a secondary role of the substrate, with starting conditions being more significant, as is expected of a chaotic system.

### 4.2 Paired reentries in the cardiac arrhythmia literature

While previous theoretical studies confirmed that the Index Theorem applies to AF (Marcotte and Grigoriev, 2017; Gurevich and Roman, 2019; DeTal et al., 2022; Arno et al., 2024), they had limitations that we address here, generalizing their findings. The authors in Marcotte et al. (Marcotte and Grigoriev, 2017) demonstrated that wavelets must be bounded by pairs of opposite singularities, but did not analyze how the singularities would interact with non-conductive boundaries. Gurevich et al. (Gurevich and Roman, 2019) claimed the total topological charge is conserved, except during boundary interactions–however, as showed here, by computing the boundary’s topological charge, this inconsistency is easily solved. By generating additional wavelets to induce collisions between singularities of opposite chirality, DeTal et al. (2022) were able to effectively eliminate all rotational activity. While still considering the existence of “single” rotors, they demonstrated that such rotors could be eliminated by treating the boundary as a mirror, effectively connecting each rotor to its “mirror image” with opposite chirality. This approach, while effective for generating opposite wavelets, still assumes that the total topological charge is not conserved when dealing with boundaries. Here, we show that, in fact, by simply considering obstacles as also being able to host reentries, the inconsistency is easily patched, and the Index Theorem is preserved. Arno et al. (2024), somewhat as a side result, showed that the Index Theorem must hold, but did not examine boundary interactions, as their interest was largely on meandering rotors with linear cores.

Moreover, because all of these works are focused on theoretical developments, their results, both simulated and experimental, are shown in rather small datasets, seeking to illustrate more than demonstrate. The data itself was either simulated in 2D tissue or experimentally observed from cell cultures. In this work, we sought to overcome this with a large dataset that incorporates clinically-derived anatomical and structural information, showing both functional and anatomical reentries can be treated in the same way.

Perhaps even more notably, it is still common in clinical work to describe the presence of both “single” and “paired” rotors (Lin et al., 2013; Narayan et al., 2014; Nattel et al., 2017; Rappel et al., 2024). Although theoretical knowledge has existed for years, albeit with limitations, it has not yet been widely spread in the clinical side of the field. We noted the same in our previous AT work (Duytschaever et al., 2024; Abeele et al., 2025), with clinicians often not making ablations that connected all singularities, and therefore unknowingly risking the possibility of arrhythmia reoccurrence. While our own simulated ablation strategy is not realistically applicable, it gives an indication towards the paths that should be taken in future developments.

In our previous AT work, we identified boundaries as of 3 types: NCB, CB1, and CB2 (Duytschaever et al., 2024; Abeele et al., 2025). We correctly identified that CB1 and CB2, the boundaries that host reentries, must be connected by an ablation line to terminate the reentrant arrhythmia. With the context of this work, we can refine that understanding. As we showed here, simply connecting opposite singularities does not guarantee termination, and is highly dependent on the particular choice of connection. While it eliminates the currently present singularities, the possibility that new singularities may emerge later means that the arrhythmia may reoccur. In our previous work, as long as all opposite singularities were connected, we could expect termination–thanks to the characteristic regularity of AT, which guarantees that eventually all waves would collide against the ablation lines and have nowhere to propagate, terminating all activity. This continues to be true, as demonstrated in initial clinical trials (Duytschaever et al., 2024), but it is a property of AT that cannot be extended to AF. That is why in this work we tested both the geodesic line virtual ablation, and the heuristic wavefront-based virtual ablation, showing that termination is only certain if conduction block not only connects two opposite singularities, but also makes it impossible for new singularities to arise later. Our chosen approach was a “brute force” upper limit to demonstrate the concept, but future work may investigate a way to optimize ablation configurations, and possibly test more realistic implementations.

### 4.3 Limitations

Our dataset consisted exclusively of simulated data, as our analysis would require global simultaneous AF data at a resolution that is not currently clinically available. Future works may use different models, or experimental data, to further verify our observations.

The presence of functional reentries in clinical practice remains controversial, as they have so far been primarily reported in computational studies (Xu et al., 2023; Allessie and de Groot, 2014; Waks and Josephson, 2014). Consequently, our findings are mainly relevant in the context of AF maintained by rotational activity. Nevertheless, because most computational studies on AF describe reentries as the dominant sustaining mechanism, our work directly contributes to this line of research (Narayan et al., 2014; Morgan et al., 2016; Vigmond et al., 2016; Haissaguerre et al., 2014).

As is common in simulation studies, we aimed to ensure physiological realism by incorporating MRI-derived atrial geometries and fiber orientations, and by employing the Courtemanche model, which captures key electrophysiological properties of human atrial tissue. Within this framework, we observed the emergence of multiple reentries, both stable and unstable.

Our chosen induction method, based on OpenCARP’s phase distribution approach, is less realistic than stimuli delivered by either the cardiac conduction system or from pacing during treatment. However, as we showed with our complexity measures, the majority of simulations evolved dynamically over time in organic ways that distinguish it from the initial setup.

Our virtual ablation strategy is not clinically realistic, as we instantly create conduction block lines, which in a real setting would move over the time needed to ablate. Moreover, our observations relate only to acute termination, and we have not verified re-inducibility or long-term effects. However, that was not the focus of this study. Rather, this is meant as a proof of concept to demonstrate the importance of the Index Theorem for our AF model, and our assertion of how connecting singularities is necessary, but not sufficient. It is not a viable recommendation, but rather a demonstration of issues with the current understanding of how to achieve termination through ablation.

As previously mentioned, while having a high performance, our algorithm could not detect an index sum of zero for all times. This was largely due to issues with clustering, and brief failures to detect phase jumps. While these errors do not significantly affect our observations and conclusions, we aim to introduce a future work with an updated version of the method that identifies singularities more robustly.

Our simulations cover common proposed AF mechanisms, with a combination of anatomical reentries, rotors, and meandering wavelets, but do not consider every proposed AF mechanism. For instance, focal activity was not simulated in our data, as it tends to terminate if its source is ablated. For example, the pulmonary veins, a common source of focal activity, especially in paroxysmal AF, would likely still require dedicated ablation even if our virtual ablation lines were clinically feasible.

The epi-endo dissociation mechanism was also not examined, since our work focuses on modeling the atrium as a surface of negligible depth. That mechanism requires each atrium to be treated as at least two surfaces with communication channels between them, or possibly even as a more complex 3D structure with volume.

Finally, we do not consider bi-atrial arrhythmias, which have similarities with the epi-endo case, as the two atria can be modeled as two surfaces with connecting channels between them. We are currently developing a new study that focuses on that topic.

## 5 Conclusion

This work demonstrated that much like in AT, the Index Theorem must also hold for simulated AF, using a large and realistic dataset of patient-based simulations, which brings more insight to the mechanisms of AF. We also showed that virtual ablation that simply connects opposite-chirality singularities may not terminate AF, highlighting issues with current ablation strategies. The greater aim was to guide future clinical work, dispelling misconceptions about functional reentries and guiding future ablation methods.

## Data Availability

The raw data supporting the conclusions of this article will be made available by the authors, without undue reservation.

## References

[B1] Abeele R. V. D. Hendrickx S. Carlier N. Wülfers E. M. Santos Bezerra A. Verstraeten B. (2025). DGM-TOP: automatic identification of the critical boundaries in atrial tachycardia. Front. Physiology 16, 1563807. 10.3389/fphys.2025.1563807 PMC12149188 40496247

[B2] Allessie M. de Groot N. (2014). CrossTalk opposing view: rotors have not been demonstrated to be the drivers of atrial fibrillation. J. physiology 592 (15), 3167–3170. 10.1113/jphysiol.2014.271809 25085969 PMC4146363

[B3] Arno L. (2021). “Phase defect lines during cardiac arrhythmias: from theory to experiment,” in arXiv preprint arXiv:2101.00315.

[B4] Arno L. Kabus D. Dierckx H. (2024). Analysis of complex excitation patterns using Feynman-like diagrams. Sci. Rep. 14, 28962. 10.1038/s41598-024-73544-z 39578507 PMC11584638

[B5] Atienza F. Climent A. M. Guillem M. S. Berenfeld O. (2015). Frontiers in non-invasive cardiac mapping: rotors in atrial fibrillation-body surface frequency-phase mapping. Card. Electrophysiol. Clin. 7 (1), 59–69. 10.1016/j.ccep.2014.11.002 25729463 PMC4341909

[B6] Courtemanche M. Ramirez R. J. Stanley N. (1998). Ionic mechanisms underlying human atrial action potential properties: insights from a mathematical model. Am. J. Physiology-Heart Circulatory Physiology 275 (1), H301–H321. 10.1152/ajpheart.1998.275.1.h301 9688927

[B7] DeTal N. Kaboudian A. Fenton F. H. (2022). Terminating spiral waves with a single designed stimulus: teleportation as the mechanism for defibrillation. Proc. Natl. Acad. Sci. 119, e2117568119. 10.1073/pnas.2117568119 35679346 PMC9214532

[B8] Duytschaever M. Van den Abeele R. Carlier N. Bezerra A. S. Verstraeten B. Lootens S. (2024). Atrial topology for a unified understanding of typical and atypical flutter. Circulation Arrhythmia Electrophysiol. 17, e013102. 10.1161/CIRCEP.124.013102 39498566

[B9] Duytschaever M. De Smet M. Martens J. El Haddad M. De Becker B. Francois C. (2025). How a topological mindset may offer extra control during mapping and ablation of left-sided reentrant atrial tachycardia. Circulation Arrhythmia Electrophysiol. 18 (7), e013780. 10.1161/CIRCEP.125.013780 40510013

[B10] Gurevich D. R. Grigoriev R. O. (2019). Robust approach for rotor mapping in cardiac tissue. Chaos An Interdiscip. J. Nonlinear Sci. 29, 5. 10.1063/1.5086936 31154775 PMC6499622

[B11] Haïssaguerre M. Sanders P. Hocini M. Hsu L. F. Shah D. C. Scavée C. (2004). Changes in atrial fibrillation cycle length and inducibility during catheter ablation and their relation to outcome. Circulation 109 (24), 3007–3013. 10.1161/01.CIR.0000130645.95357.97 15184286

[B12] Haissaguerre M. Hocini M. Denis A. Shah A. J. Komatsu Y. Yamashita S. (2014). Driver domains in persistent atrial fibrillation. Circulation 130 (7), 530–538. 10.1161/CIRCULATIONAHA.113.005421 25028391

[B13] Lee S. Sahadevan J. Khrestian C. M. Cakulev I. Markowitz A. Waldo A. L. (2015). Simultaneous biatrial high-density (510–512 electrodes) epicardial mapping of persistent and long-standing persistent atrial fibrillation in patients: new insights into the mechanism of its maintenance. Circulation 132 (22), 2108–2117. 10.1161/CIRCULATIONAHA.115.017007 26499963 PMC4666790

[B14] Lin Y.-J. Lo M. T. Lin C. Chang S. L. Lo L. W. Hu Y. F. (2013). Prevalence, characteristics, mapping, and catheter ablation of potential rotors in nonparoxysmal atrial fibrillation. Circulation Arrhythmia Electrophysiol. 6, 851–858. 10.1161/CIRCEP.113.000318 23983246

[B15] Lootens S. Janssens I. Van Den Abeele R. Wülfers E. M. Bezerra A. S. Verstraeten B. (2024). Directed graph mapping exceeds phase mapping for the detection of simulated 2D meandering rotors in fibrotic tissue with added noise. Comput. Biol. Med. 171, 108138. 10.1016/j.compbiomed.2024.108138 38401451 PMC10966475

[B16] Marcotte C. D. Grigoriev R. O. (2017). Dynamical mechanism of atrial fibrillation: a topological approach. Chaos An Interdiscip. J. Nonlinear Sci. 27, 093936. 10.1063/1.5003259 28964130

[B17] Maury P. Takigawa M. Capellino S. Rollin A. Roux J. R. Mondoly P. (2019). Atrial tachycardia with atrial Activation Duration Exceedang the Tadhycardia Cycle Length: Mechenisms and Prevalente. cACC Clin. Electrophysiol. 5 (8), 907–916. 10.1016/j.jacep.2019.04.015 31439291

[B18] Morgan R. Colman M. A. Chubb H. Seemann G. Aslanidi O. V. (2016). Slow conduction in the border zones of patchy fibrosis stabilizes the drivers for atrial fibrillation: insights from multi-scale human atrial modeling. Front. Physiology 7, 474. 10.3389/fphys.2016.00474 27826248 PMC5079097

[B19] Nagy S. Z. Kasi P. Afonso V. X. Bird N. Pederson B. Mann I. E. (2021). Cycle Length Evaluation in Persistent Atrial Fibrillation Using Kernel Density Estimation to Identify Transient and Stable Rapid Atrial Activity. Cardiovasc. Eng. Technol. 13 (2), 219–233. 10.1007/s13239-021-00568-1 34453278 PMC9114079

[B20] Narayan S. M. Baykaner T. Clopton P. Schricker A. Lalani G. G. Krummen D. E. (2014). Ablation of rotor and focal sources reduces late recurrence of atrial fibrillation compared with trigger ablation alone: extended follow-up of the CONFIRM trial (Conventional Ablation for Atrial Fibrillation With or Without Focal Impulse and Rotor Modulation). J. Am. Coll. Cardiol. 63 (17), 1761–1768. 10.1016/j.jacc.2014.02.543 24632280 PMC4008643

[B21] Nattel S. Xiong F. Aguilar M. (2017). Demystifying rotors and their place in clinical translation of atrial fibrillation mechanisms. Nat. Rev. Cardiol. 14, 509–520. 10.1038/nrcardio.2017.37 28383023

[B22] Plank G. Loewe A. Neic A. Augustin C. Huang Y. L. Gsell M. A. F. (2021). The openCARP Simulation Environment for Cardiac Electrophysiology. Under Rev. bioRxiv Prepr. 208, 106223. 10.1016/j.cmpb.2021.106223 34171774

[B23] Rappel W.-J. Baykaner T. Zaman J. Ganesan P. Rogers A. J. Narayan S. M. (2024). Spatially Conserved Spiral Wave Activity During Human Atrial Fibrillation. Circulation Arrhythmia Electrophysiol. 17, e012041. 10.1161/CIRCEP.123.012041 38348685 PMC10950516

[B24] Roney C. H. Sim I. Yu J. Beach M. Mehta A. Alonso Solis-Lemus J. (2022). Predicting Atrial Fibrillation Recurrence by Combining Population Data and Virtual Cohorts of Patient-Specific Left Atrial Models. Circulation Arrhythmia Electrophysiol. 15.2, e010253. 10.1161/CIRCEP.121.010253 35089057 PMC8845531

[B25] Santucci P. A. Bhirud A. Vasaiwala S. C. Wilber D. J. Green A. (2024). Identification of 2 Distinct Boundaries Distinguishes Critical From Noncritical Isthmuses in Ablating Atypical Atrial Flutter. Clin. Electrophysiol. 10.2, 251–261. 10.1016/j.jacep.2023.09.024 37999671

[B26] Tomii N. Yamazaki M. Ashihara T. Nakazawa K. Shibata N. Honjo H. (2021). Spatial phase discontinuity at the center of moving cardiac spiral waves. Comput. Biol. Med. 130, 104217. 10.1016/j.compbiomed.2021.104217 33516959

[B27] Vandersickel N. (2024). Impact of topology on the number of loops during macro-re-entrant atrial tachycardia. 10.1093/eurheartj/ehae032 38339877

[B28] Vandersickel N. Hendrickx S. Van Den Abeele R. Wuelfers E. Bezerra A. S. Fuenmayor S. (2023). Unique topological classification of complex reentrant atrial tachycardias enables optimal ablation strategy. Europace 25, euad122.078. 10.1093/europace/euad122.078

[B29] Vigmond E. Pashaei A. Amraoui S. Cochet H. Hassaguerre M. (2016). Percolation as a mechanism to explain atrial fractionated electrograms and reentry in a fibrosis model based on imaging data. Heart Rhythm 13 (7), 1536–1543. 10.1016/j.hrthm.2016.03.019 26976038

[B30] Waks J. W. Josephson M. E. (2014). Mechanisms of Atrial Fibrillation – Reentry, Rotors and Reality. Arrhythmia and Electrophysiol. Rev. 3.2, 90–100. 10.15420/aer.2014.3.2.90 26835073 PMC4711504

[B31] Wartenberg D. (1985). Multivariate Spatial Correlation: A Method for Exploratory Geographical Analysis. Geogr. Anal. 17, 263–283. 10.1111/j.1538-4632.1985.tb00849.x

[B32] Xu C.-H. Xiong F. Jiang W.-F. Liu X. Liu T. Qin M. (2023). Rotor mechanism and its mapping in atrial fibrillation. Europace 25 (3), 783–792. 10.1093/europace/euad002 36734272 PMC10062333

